# Breastfeeding and COVID-19: From Nutrition to Immunity

**DOI:** 10.3389/fimmu.2021.661806

**Published:** 2021-04-07

**Authors:** Emilia Vassilopoulou, Gavriela Feketea, Lemonica Koumbi, Christina Mesiari, Elena Camelia Berghea, George N. Konstantinou

**Affiliations:** ^1^Department of Nutritional Sciences and Dietetics, International Hellenic University, Thessaloniki, Greece; ^2^PhD School, “Iuliu Hatieganu” University of Medicine and Pharmacy, Cluj-Napoca, Romania; ^3^Department of Pediatrics, Pediatric Allergy Outpatient Clinic, “Karamandaneio”, Children Hospital, Patras, Greece; ^4^Department of Pediatrics, Allergology and Clinical Immunology Outpatient Clinic, Clinical Hospital of Emergency for Children MS Curie, Bucharest, Romania; ^5^Department of Pediatrics, Carol Davila University of Medicine and Pharmacy, Bucharest, Romania; ^6^Department of Allergy and Clinical Immunology, 424 General Military Training Hospital, Thessaloniki, Greece

**Keywords:** antibodies, anti-inflammation, breastfeeding, COVID-19, SARS-CoV-2, microbiome, virome, respiratory infection

## Abstract

Breastfeeding not only provides the optimum source of nutrients for the neonate and its first strong shield against infection but also lays the foundation for somatic and psychological bonding between the mother and child. During the current COVID-19 pandemic, although the guidelines of the relevant international and national agencies recommend breastfeeding by SARS-CoV-2–infected mothers, considerable insecurity persists in daily clinical practice regarding the safety of the infants and the perceived advantages and disadvantages of discontinuation of breastfeeding. This is a systematic review of the currently available information regarding the transmissibility of SARS-CoV-2 through or while breastfeeding and the protection against infection that breast milk might provide. The accumulated body of knowledge regarding the role of breast milk in the development of the neonatal immune system and protection against infection by other respiratory viruses is discussed, with a focus on the anti-inflammatory role of the antibodies, microbes, and viruses provided to the infant in breast milk and its relevance to the case of SARS-CoV-2.

## Highlights

- Nature has designed the mother’s breast milk to nurture the neonate and to protect of the dyad in “psyche and soma”, ensuring the transfer of antibodies, microbes, and viruses, but also emotional stimulation.
- Since SARS-CoV-2 is highly transmissible, breastfeeding should be encouraged, but observing all appropriate safety measures, for the mother and close contacts
- With the vaccination schedule in progress, the pregnant mothers-to-be should probably be prioritized.

## Brief Historical Perspective

Since the beginning of 2020, when the World Health Organization (WHO) announced a new strain of coronavirus, the SARS-CoV-2, which provokes coronavirus disease 19 (COVID-19) (1), the whole world has been dominated by the COVID-19 pandemic. In January 2021, only 1 year later, approximately 85 million cases had been confirmed, resulting in more than 1.8 million deaths (2).

During the same period, approximately 140 million births have been registered (3) and a great dilemma arose regarding the possible need to discontinue the breastfeeding of infants of infected mothers (4). Although, to date, evidence on the risk of vertical transmission, *via* the respiratory tract or through the breast milk itself, is limited, breastfeeding has generally been accepted as the preferred nutrition for the infant of the infected mother. In breast milk from infected mothers, IgA antibodies against SARS-CoV-2 have been detected, which may account for the reduced clinical impact of the disease in breastfed infants upon future viral exposure (5).

This mini-review summarizes the current evidence, collected in a systematic literature search and narrative review of original articles related to breastfeeding by SARS-CoV-2–infected mothers, and deploying knowledge gained from other respiratory virus-transmitted diseases about the protective anti-inflammatory effects of breastfeeding. The specific questions addressed in the review were whether or not the infant should be breastfed when (1): the mother is diagnosed with SARS-CoV-2 before or immediately after delivery; (2) the lactating mother is positive for SARS-CoV-2 but the infant is negative; (3) both mother and infant are positive for SARS-CoV-2; (4) or the mother is negative but the infant is positive for SARS-CoV-2, based on evidence from the literature and knowledge gained from other respiratory virus infections.

## Methods

The review followed the Preferred Reporting of Systematic Reviews and Meta-Analysis (PRISMA) guidelines to collect articles on breastfeeding by SARS-CoV-2–infected mothers (6). This review was not registered with PROSPERO, which does not currently accept registration of reviews.

We searched the PubMed, Scopus, Web of Science, and MedRxiv electronic databases up to December 31^st^ 2020 to identify original published studies describing lactating women with a confirmed diagnosis of COVID-19, using the following search keywords and phrases: (“COVID-19” OR “2019-nCoV” OR “novel coronavirus” OR “SARS-CoV-2” OR “coronavirus 2”) AND breastfeeding. Reference tracking was carried out to identify other studies eligible for inclusion.

Each reference retrieved was screened independently by two researchers, following predefined criteria to determine eligibility for the systematic review. Studies were excluded if: 1. they did not involve humans (e.g., *in vitro* or animal research), and 2. they were non-original articles (e.g., book chapters, review articles, metanalysis, guidelines). There were no date or language restrictions on the search.

All the original observational studies, case reports and case series of breastfeeding women diagnosed with COVID-19 identified in the search were included. In view of the heterogeneity observed across the studies, it was decided to perform a narrative synthesis, using the Synthesis Without Meta-analysis (SWiM) reporting guidelines (intended to complement the PRISMA guidelines) (7). Descriptive statistics were presented (frequency and proportions) based on the total cases with available information. In addition, the current guidelines issued by international and national health organizations were retrieved and summarized. Documentation on other maternal respiratory viral infections and breastfeeding was gathered and the evidence on the potential protective effect of breastfeeding for the infants is presented and discussed.

## Summary of the Established Principles

### Current Recommendations About Breastfeeding and COVID-19

Human breastfeeding enhances both maternal and infant health, with a dynamic, bidirectional exchange between the mother and the infant, which constitutes the cornerstone of infant and child well-being.

Despite concerns of transmission from the infected mother to the infant, global and national health stakeholders have so far univocally encouraged breastfeeding during the COVID-19 pandemic. The WHO (8), the United Nations International Children’s Emergency Fund (UNICEF) (9), the Union of European Neonatal & Perinatal Societies (UENPS) (10), and the US Centers for Disease Control and Prevention (CDC) (11), all highlight the well-established overall short- and long-term immunological and psychosomatic benefits of breastfeeding for the dyad. The current recommendations point out that there is, at present, insufficient evidence about the transmission of COVID-19 through breastfeeding. For this reason, strict measures of mother-infant separation and discontinuation of breastfeeding are to be avoided, regardless of a positive diagnosis and the intensity of symptoms, unless the severity is of such a level that the mother cannot take care of the infant, in which case, expressed, fresh, unpasteurized breast milk should be provided for the baby (8).

In spite of these guidelines, there has considerable scepticism during the pandemic among the front-line healthcare professionals, gynecologists, midwives, and pediatricians, on whether they should encourage the infected mother to breast feed her baby (12).

## Studies Related to Breastfeeding of SARS-Cov-2–Infected Mothers

**Figure 1:**
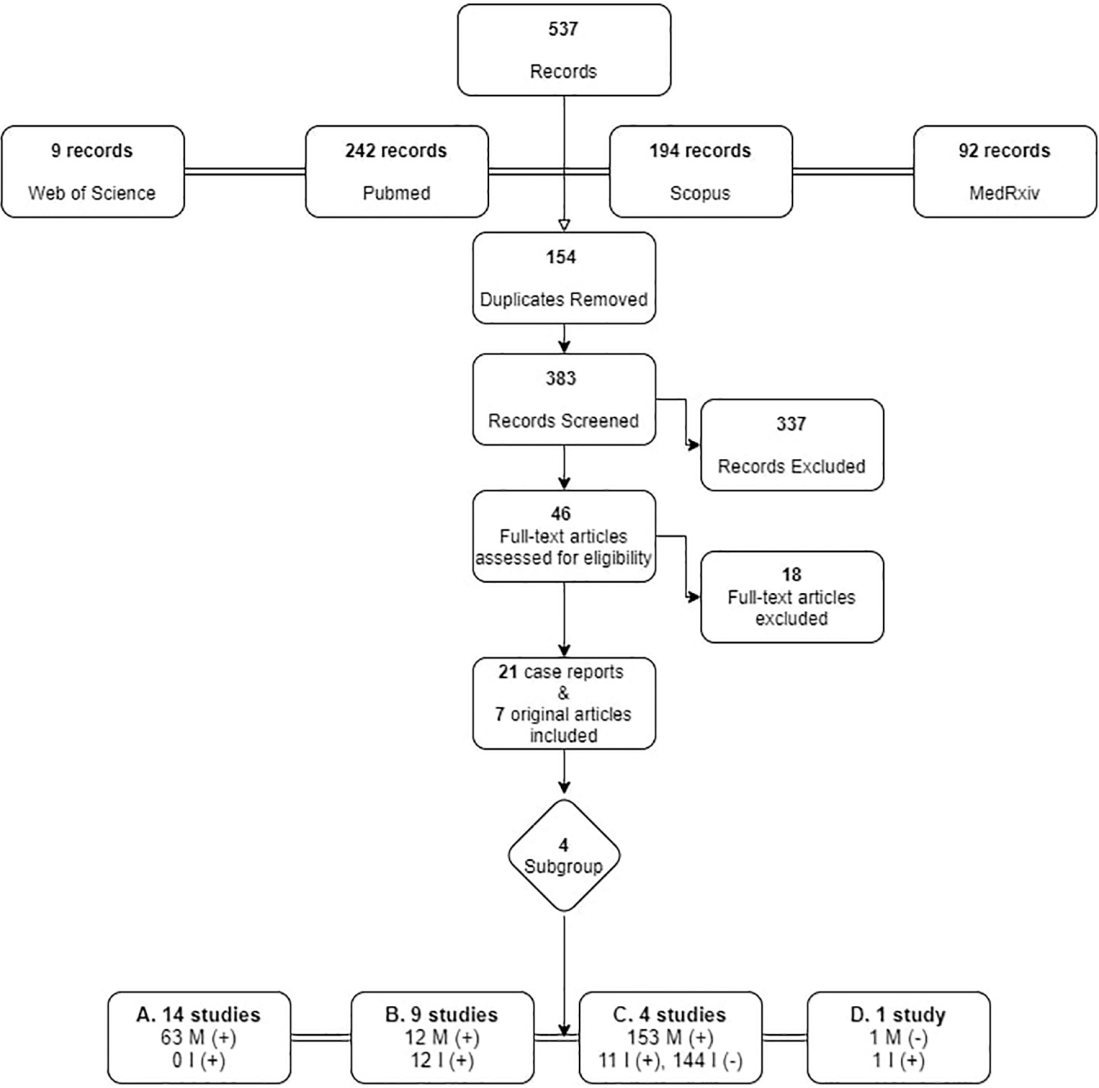
Search strategy flowchart for original articles on breastfeeding and SARS-Cov-2. M, Mother; I, Infant. +: SARS-CoV-2 infected, -: negative for SARS-CoV-2.

From the search in the four electronic databases, 537 articles were retrieved, and after removal of duplicates, 383 were screened. Among 46 articles reaching the final assessment for eligibility, 18 were excluded, as they did not meet all inclusion criteria, i.e., they did not provide data on whether the mother and/or infant was infected, whether the infant was breastfeeding and what practices were used. Finally, 21 case reports and 7 original articles, published up until December 31^st^ 2020 were included, originating mainly from China (11 case reports; 1original article) and Italy (2 case reports; 2 original articles). **Figure 1** presents the process of inclusion of studies in the review.

**Graphical Abstract:**
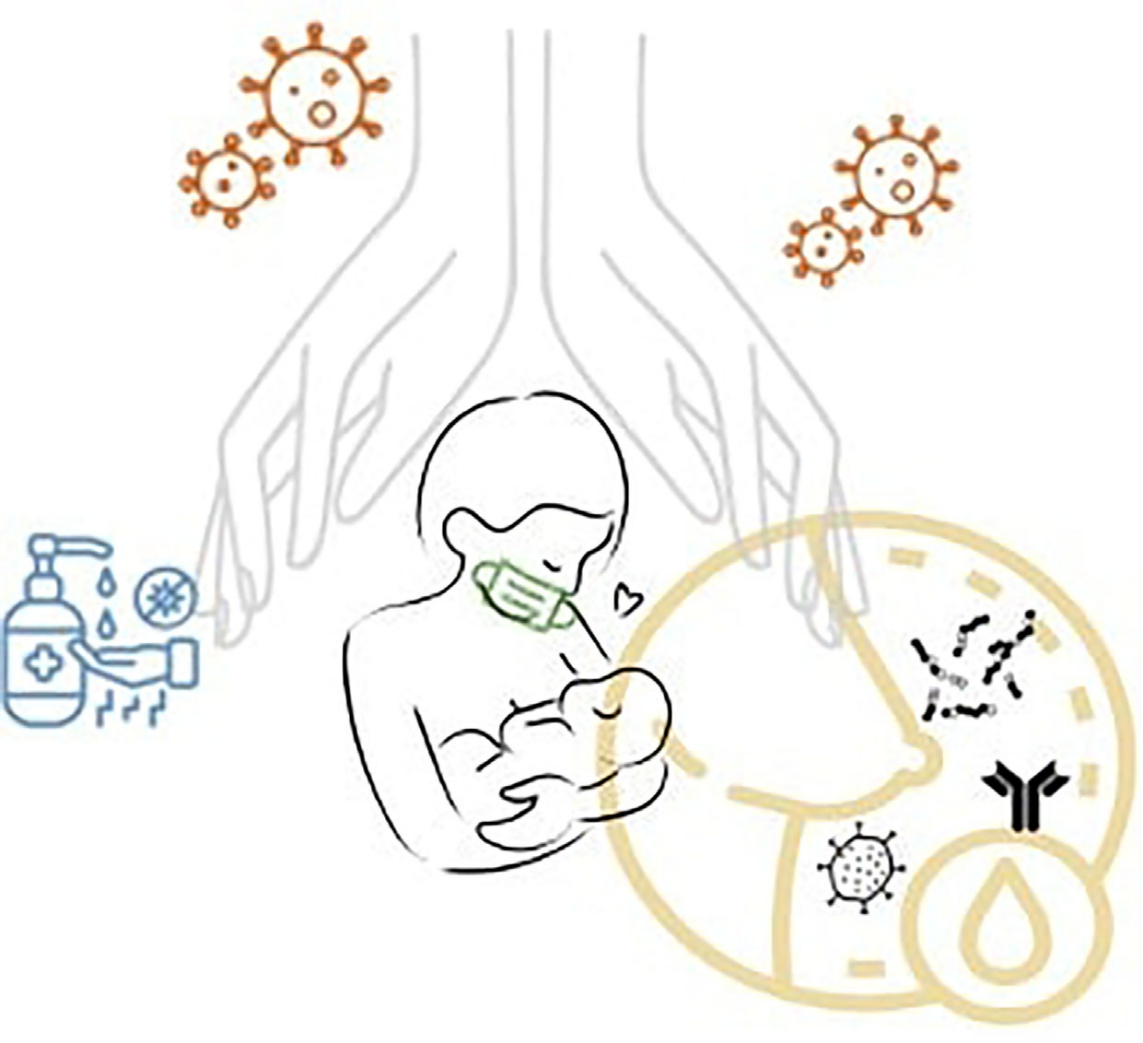


For purposes of this review the studies were sub-grouped into four categories according to the infected member of the dyad, and the measures undertaken are discussed accordingly. A summary of the 28 studies is presented in **Table 1**.

**Table 1:** Studies included in the review of breastfeeding (BF) and SARS-CoV-2 infection (N=28).

Authors	Year	Country	Study Type	No of infected mothers	Mother’s age (years)	Infant’s Age (months)	Mother’s Symptoms	Infant’s Symptoms/test	Feeding mode	Maternal Separation (Y, yes/N, no)	SARS-CoV-2 in breastmilk	Conclusion
**A. Infected mother–non-infected infant**												
**Lowe and Bopp** (13)	2020	Australia	Case report	1	31	<1	Fever & respiratory symptoms	(−)	BF	N	N/A	BF & room-in with precautions
**Pereira et al**. (14)	2020	Spain	Retrospective case series	22	34 (19–43)	<1	50% symptomatic (6 mild symptoms, 5 pneumonia)	(−)	20/22 BF	9/11 symptomatic mothers isolated	N/A	BF with precautions, Upon maternal isolation: feeding with pasteurized DHM or IF until BF is resumed
									2/22 IF			
**Lang and Zhao** (15)	2020	China	Case report	1	30	<1	Dry cough	(−)	BF after isolation	Y	(−)	BF after isolation and negative test, PBM during isolation
**Bastug et al**. (16)	2020	Turkey	Case report	1	20	<1	Asymptomatic	(−)	PBF, When SARS-CoV-2 detected in BM-BF discontinued	Y	(+)	Decision about BF by parent & doctor
**Gabriel et al**. (17)	2020	Spain	Observational prospective study	7	33.4 (31–37)	<1	6/7 asymptomatic 1/7 fever, myalgia, malaise, headache	(−)	BF	N/A	(−)	Direct BF safe or hand: expressed BM with precautions
**Chu et al**. (18)	2020	China	Case report	1	30	<1	Gastrointestinal symptoms, fever	(−)	PBM	Y	(−)	SARS-CoV-2 rarely transmitted through BM- possible induction of passive immunity *via* lgG
**Liu et al**. (19)	2020	China	Case report	1	37	6	Fever, cough, sore throat, fatigue	(−)	BF discontinued after mother’s positive test	Y	N/A	PBM and feeding to infant by a healthy caretaker/family member
**Feng et al**. (20)	2020	China	Case report	1	33	2	Fever	(−)	Suspended BF after diagnosis	Y	N/A	BF temporal suspension
**Perrone et al**. (21)	2020	Italy	Case report	1	N/A	<1	fever, anosmia, & malaise	(−)	BF + expressed maternal milk	N	(−)	BF with precautions when virus is not traced in milk
**Liu et al**. (22)	2020	China	Prospective study	19	31 (27–34)	<1	fever (11/19), 5/19 cough or dyspnea, 2/19 diarrhea or another gastrointestinal symptom	(−)	Term Formula	Y	(−)	Not vertical transmission with BF
**Dong et al**. (23)	2020	China	Case report	1	33	<1	Cough & chest tightness	(−)	BF	Y	(−)	lgG & lgA of BM might provide immune protection
**Zhu et al. (**24**)**	2020	China	Case report	5	32 (27–34)	<1	Main symptoms: fever, chest distress or dyspnea, cough, nasal congestion, rhinorrhea, poor appetite, or diarrhea	(−)	N/A	N/A	1/5 (+)	Generally BF encouragement but when SARS-CoV-2 BM increased risk of transmission
**Dong et al. (**25**)**	2020	China	Case report	1	29	<1	Fever, nasal congestion, respiratory difficulties	(−)	N/A	Y	(−)	When increased lgG & lgM levels-infection at delivery cannot ruled out
**Li et al**. (26)	2020	China	Case Report	1	30	<1	Dry cough, fever	(−)	N/A	Y	(−)	mother-to-child transmission is unlike.
**B. Infected mother–Infected infant**												
**Yu et al**. (27)	2020	China	Case report	1	32	13	Nasal congestion	(+)	Directly BF & complementary after 6 months	N	(−)	BF safe
								Fever, dry cough, nasal congestion				
**Groß et al**. (28)	2020	Germany	Case series	2	N/A	<1	Mild symptoms	(+)	BF	N	Mother’s 1 (−)	BF with precautions/further investigation on virus transmission potential during BF
								Respiratory, breathing symptoms icterus			Mother’s 2	
											(+)	
**Tam et al**. (29)	2020	Australia	Case series	1	40	8	Sore throat, myalgia, productive cough, fever	(+)	BF discontinued and PBM when infant was confirmed with COVID-19	N	(+)	In infected infants BF should be continued/nil adverse effects/SARS-CoV-2 RNA in breast milk sample did not indicate viable virus
								Mild coryzal symptoms, non-productive cough				
**Salvatori et al**. (30)	2020	Italy	Case report	2	31 (26–36)	<1	50% anosmia & dysgeusia, 50% back & thoracic pain	(+)	BF	N	(−)	BF supported, transmission more possibly through respiratory droplets
								50% asymptomatic				
								50% diarrhea, cough, poor feeding				
**Phadke et al**. (31)	2020	India	Case report	1	33	<1	Asymptomatic	(+)	IF	Y	N/A	BF & room-in with precautions
								asymptomatic	After positive test for infant: direct BF			
**Kirtsman et al**. (32)	2020	Canada	Case report	1	40	<1	myalgia, decreased appetite, fatigue, dry cough & fever	(+)	BF	N	(+)	Further studies to neonates are needed to identify transmission routes
								Neutropenic, mild hypothermia, feeding difficulties & hypoglycemic episodes				
**Hinojosa-Velasco et al**. (33)	2020	Mexico	Case report	1	21	<1	Cough, odynophagia, headache, diarrhea, rhinorrhea, sore throat, fever	(+) newborn jaundice, tachypnea, hypernatremia, central cyanosis, dyspnea, and oxygen saturation of less than 92%, (NICU)	Synthetic milk formula	Y	(+)	BF might be protective
**Wang et al**. (34)	2020	China	Case report	1	34	<1	Fever, vaginal bleeding, lower abdominal pain	(+)	Infant Formula	Y	(−)	No BF
**Fan et al. (**35**)**	2020	China	Case series	2	31.5 (29–34)	<1	1: Nasal congestion, fever	(+)	IF	1/2 Y	(−)	Low risk of vertical transmission *via* BF, potential protective role of passive antibodies
							2: fever, nasal congestion, sore throat	1: fever and abdominal distension with lymphopenia				
								2: mild neonatal pneumonia and lymphopenia				
**C. Infected mother- both infected and non-infected infants**												
**ElHalik et al**. (36)	2020	United Arab Emirates	Retrospective observational study	35	32 (24–42)	<1	Negative	34/36 (−)	32/36 direct BF or PBM	27/36 roomed-in	N/A	Direct BF or PBM encouraged with precautions
								2/36 (+)				
								1 asymptomatic				
								1 admitted to NICU with respiratory distress				

**Ronchi et al**. (37)	2020	Italy	Prospective multicenter study	61	32 (28–36)	<1	Symptoms at diagnosis: 34/61 asymptomatic	0/62 on birth	45/62 Exclusive BF	51/52 roomed-in exclusive BF	N/A	BF and room-in encouraged to infected mothers with good clinical condition with precautions
							27/61 at least 1 symptom	2/62 positive for SARS-CoV-2 at 7 and 20d of life respectively	13/62 BF + IF 3/62 exclusive IF 1/62 BF + PBM			
**Patil et al**. (38)	2020	United States of America	Retrospective cross-sectional study	45	30 (27.5–35.5)	<1	27/45 asymptomatic	42/45 (+) SARS-CoV-2	31/33	33/45 roomed-in	No tested sample	BF and room-in under proper precautions
							16/45 mild symptoms	3/45 -(+)/admitted to NICU-asymptomatic		5/45 separated,		
							2/45 ICU			7/45 preterm admitted to NICU		
		
**Bertino et al**. (39)	2020	Italy	Prospective Collaborative Observational Study	12	30.5 (24–38)	<1	10/12 symptomatic: fever, myalgia, rhinitis, cough, dyspnea, sore throat, conjunctivitis, diarrhea, chest pain, anosmia, ageusia	4/12 (+) SARS-CoV-2 asymptomatic	11/12 exclusively BF	No	11/12 (−)	BF or PBM (in isolation) encouraged irrespective swab test results with proper precautions
											1/12 (+)	
**D. Non-infected mother-infected infant**												
**Le et al**. (40)	2020	Vietnam	Case report	1 (non-infected)	N/A	3	None (−)	(+) Rhinorrhea, nasal congestion & fever	Exclusively BF	No	No tested sample	Need to further investigate SARS-CoV-2 transmission routes to pediatric population

BF, breastfeeding; IF, infant formula; Y, yes; n, no; N/A, not available; DHA, donor human milk; PBM, pumped breast milk; PBF, pumped breastfeeding; BM, breast milk; (−), negative; (+), positive; NICU, neonatal intensive care unit.

### SARS-CoV-2–Positive Mother Before or on Delivery

Some mothers were prohibited from holding the neonates, in order to minimize the risk of infection (36, 39) and expressed breast milk was proposed as the optimum feeding solution (38). Mothers in a good clinical condition were encouraged to breastfeed, with all appropriate instructions and precautions (37). In the case of admission of preterm infants to the neonatal intensive care unit (NICU), expressed breast milk was suggested, if available (36). Overall, among the 231 births from SARS-CoV-2–positive mothers, 13 neonates (5.8%) tested positive within the first 48 hours of life (31–34, 36, 38, 39).

### SARS-CoV-2–Positive Lactating Mother, With Negative Infant

Generally, breastfeeding was encouraged (13, 14, 17, 23, 24, 26), but in order to reduce the risk of infection (36, 39), instructions for appropriate precautions were given (37). Measures undertaken to minimize the risk of transmission during breastfeeding were mask wearing, handwashing, routine cleaning and disinfection of all surfaces touched, thorough cleaning and sterilization of infant feeding equipment before and after use, breast washing with gauze saturated with soap and water, and avoidance of falling asleep with the baby (13, 14, 17). Alternatively, expression of breast milk and feeding of the infant by a healthy family member or a caretaker was recommended (19). In some cases, when feeding became a controversial issue, the parents were to allowed decide if the infant would be breast fed, due to the insecurity caused by the perceived risk of transmission to the infant (16, 22). Insecurity about the decision was increased when SARS-CoV-2 was traced in the breast milk, in which case, breastfeeding was suspended and could be resumed after a period of isolation, followed by a confirmed negative test on the mother (15, 20). Of a total of 63 women, 37 (58.7%) discontinued breastfeeding and were isolated from their babies. Of over 38 samples of breast milk tested in SARS-CoV-2–positive mothers, only 2 (5.2%) were positive for SARS-CoV-2 (16, 24).

### Both Mother and Infant SARS-CoV-2–Positive

Breastfeeding was strongly encouraged where both members of the dyad were positive (27, 30, 31), and further investigation was suggested on the role of IgG antibodies (27) in the possible protective role of breast milk antibodies on the immunity of the infants (33, 35). Among the breast milk samples tested for SARS-CoV-2, 27% (3/11) were positive (27, 28, 30, 34, 35), but the milk was not considered to be the cause of infection of the infants (29).

## Healthy Mother and Infant SARS-CoV-2–Positive Infant

In the one reported case of an infected infant with a healthy mother, the mother was encouraged to remain in quarantine with her baby and to continue breastfeeding. Despite the close contact, the mother remained negative for SARS-CoV-2 (40).

## The Current “State of the Art”

### The Anti-Inflammatory Effects of Breastfeeding: Knowledge Gained From Other Respiratory Viruses

Respiratory infections are a leading cause of morbidity in children. During the first year of life, breastfeeding provides protection from these infections, dependent on its duration (41), mainly against lower respiratory infection. The immaturity of the infant’s immune system at birth increases the risk of infection by external agents, including viruses and bacteria (42), which is related to the unpreparedness of the neonatal respiratory and gastrointestinal (GI) tracts to resist invasion.

Breast milk changes in synthesis, from colostrum through a transitional stage to mature milk, ensuring appropriate nutrition for the infant (43). Intensive, continuing research has provided an appreciable body of documentation on the health benefits of breastfeeding for both mother (44, 45) and child. Regarding immunity, human milk induces in the infant the regulation and development of the innate (46) and adaptive immune systems (47, 48), with a major long-term role in health and disease (49).

The anti-inflammatory protection conveyed by breast milk is effected by both chemical components and cellular interactions. Colostrum and transitional milk safeguard the infant *via* an abundant glycoprotein, lactoferrin, which has multilevel actions, lymphostimulatory, anti-inflammatory, anti-bacterial, anti-viral and anti-fungal (50). Its protective functions are attributed to its iron-binding properties, inhibition of interleukin-1β (IL-1β) and tumor necrosis factor-alpha (TNF-α), stimulation of the activity and maturation of lymphocytes, and preservation of an antioxidant environment (51). Lactoferrin, along with other milk peptides protects against bacteria and fungus (52). *In vitro* studies have shown that lactoferrin inhibits the invasion and growth of respiratory syncytial virus (RSV), interacting directly with the F glycoprotein on the surface of the virus, which is essential for viral penetration. Adenovirus infection was also observed to be inhibited *in vitro* due to interference of lactoferrin with the primary receptors present at the cellular level. Milk regurgitation into the nose after breastfeeding has been suggested to increase phage adherence to mucosal surfaces in the respiratory tract, in addition to the gut, eliminating in this way mucosal bacteria and protecting against recurrent respiratory infections in breastfed infants, in the longer term (53).

New knowledge gained during the 2003 SARS-CoV-1 epidemic was that lactoferrin interacts with heparin sulphate glycosaminoglycan (HSPG) cell receptors, interfering with the first anchoring sites of the virus on the cell, and thus preventing the initial contact between the SARS-CoV and host cells. Lactoferrin has also been shown to block the interaction between spike viral protein and HSPC in an angiotensin-converting enzyme 2 (ACE2) receptor, which otherwise results in the full infection (54).

Other dominant oligosaccharides in human milk serve both as a direct barrier to pathogens and as a prebiotic, i.e., aliment for probiotics, which promote synthesis of a healthy microbiota (42). The binding capacity of the oligosaccharides has proven protective against viruses with high morbidity and mortality, including human immunodeficiency virus (HIV) (55) and rotavirus (56), and breast milk mucin is able to aggregate poxviruses prior to their entry into host cells (57).

Additional protective properties of breast milk are provided by the transfer of maternal immune cells to the infant, including macrophages, neutrophils and lymphocytes (58). The concentration of these cells in the human breast milk vary according to the age of the infant, taking into account that in the early stage of lactation, the neonatal immune system is completely immature (59). Thus, the proportion of the different leukocytes vary between colostrum (macrophages 40–50%, neutrophils 40–50% and lymphocytes 5–10%) and more mature milk (macrophages 85%, lymphocytes 15%).

The immune properties of the mother are also transferred to the breastfed infant in the form of secretory IgG (60) and IgA in maternal milk (61). Breast milk attains the highest concentration in IgG antibodies in the colostrum, and their concentration drops after the first month of life (62) and stops abruptly with weaning (63). IgG antibodies, transferred to the fetus through the placenta during intrauterine life and to the infant after birth in breast milk, constitute the infant’s first defense system. In mothers immunized against RSV, s-IgG antibodies were detected in breastmilk (64), providing protection to the infant against the main cause of respiratory infection during the first year of life (65). IgA antibodies coat the GI and respiratory mucosa and block the entrance of foreign antigens (50) and viruses (63). In premature infants, the IgA levels are higher, for enhanced protection (66). In the event of an infection, in either the mother or the child (67), breast milk conveys a plethora of antipathogenic and anti-inflammatory bioactive factors (68) to protect the infant. In attacks by respiratory virus infections, such as RSV, protection is mediated by polymeric IgA antibodies to a protein of the RSV surface membrane, inhibiting virus replication (50).

In addition to the transfer of antibodies, human milk triggers immune-protective responses by the host. In influenza, type I interferons (IFN), cytokines with strong simultaneous anti-viral, pro-apoptotic and pro-inflammatory properties, are produced significantly more often in breastfed infants, transforming viral attacks to *formes frustes* (69).

Breastfeeding has been documented to exert more effective protection against a spectrum of pneumonia-causing viruses, including influenza, RSV and parainfluenza, in girls than in boys (70); these findings were interpreted by the researchers as a “nature”-provided advantage for survival of females, in order to preserve the species.

Protection against viral invasion appears to be enhanced by the regurgitation of breast milk into the upper respiratory tract, conveying viable commensal, mutualistic, and probiotic bacteria (71) and viruses (53) that colonize the upper respiratory tract, contributing to the maturation of the infant’s immune system.

Bacteria in the human milk are one of the earliest sources of prokaryotic microorganisms transferred to the infant, following the maternal microbial colonization through the amniotic fluid, placenta and umbilical fluid (72), and a more substantial transmission of vaginal and gut microorganisms to the newborn through the birthing canal (73). The human milk microbiota (HMM) originates from the maternal GI tract and skin, and from the infant’s mouth (71). HMM and other human milk components, such as human milk oligosaccharides (HMO), reflect environmental factors, e.g., viral exposure, weather, and diet, in addition to the immune status of the mother (74).

Human milk transmits a significant load of viruses, eukaryotic and bacteriophages, from the mother to the infant, which enhance the maturation of both innate and adaptive immune systems. Eukaryotic viruses may impact the health status directly, while bacteriophages act *via* the bacterial ecology.

## Highlighting Future Directions in Breastfeeding Research

In children, COVID-19 infection rates are lower than in adults, while fatality rates are almost zero (71). Children generally have milder symptoms, but there are reports of the development of a novel multisystem inflammatory syndrome in children (MIS-C) similar to Kawasaki disease, predicating continuous vigilance (53).

The current policy is that breastfeeding is contraindicated in only a limited number of viral diseases, i.e., HIV, cytomegalovirus (CMV) in preterm infants, and human T-lymphotropic virus I (72). As it constitutes an incomparable feeding method for babies, international and national health authorities strongly recommend exclusive breastfeeding for at least the first six months of life: “Breastfeeding is one of the most effective ways to ensure child health and survival” - WHO (8); “Low rates and early cessation of breastfeeding have important adverse health and social implications for women, children, the community and the environment, result in greater expenditure on national health care provision, and increase inequalities in health” - European Commission (EC) (75)“.

The unsurpassed benefits of breastfeeding include not only irreplaceable nutrition, ensuring healthy growth for the infant, but also prevention of obesity in later life (74). It is a mainstay for promoting the immune development of the infant by both immunological factors transferred from mother to infant through breast milk (76) and microorganisms colonizing the organs and viruses enriching the virome.

SARS-CoV-2 is probably transmitted in multiple ways, including through droplets *via* the respiratory tract and invasion by enterocytes (73). GI symptoms manifest first in infancy, and lactoferrin in breast milk has been suggested to be capable of strengthening junctions between microbes in the gut and thus amplifying the innate defense. Although at present, this is only an assumption for SARS-CoV-2, in other strains of SARS-CoV, lactoferrin was shown to increase mucosal immunity and prevent viral anchoring on cell receptors (77). Cytokines and growth factors in breast milk excite the infant’s immune system and balance the anti-inflammatory and pro-inflammatory cytokines, lessening their effect and preventing the “cytokine storm” described in other viral infections, such as H1N1 ‘swine flu’ and H5N1 ‘bird flu’ (78).

The most abundant antibody in breast milk, sIgA, provides adequate specific protection against pathogens, among which also are viruses. The specificity of sIgA is determined by the immune response of the mother to previous infection, probably explaining the low rates of infection or milder symptoms of the infected breastfed infants of SARS-CoV–infected mothers. Concerning this phenomenon, the cases where infection is not protected against by breastfeeding should be considered. For example, in pertussis, the sIgA in the human milk of infected mothers was higher than in that of control subjects, but did not protect against infection of the infant, and therefore vaccination of pregnant women was recommended (79). The evidence to date on pregnant women infected by SARS-CoV-2 does not demonstrate a more severe or complex clinical picture than in the general infected population. Further investigation is needed to accumulate knowledge regarding anti-SARS-CoV-sIgA produced through breastfeeding for the neonate.

In view of the time-dependent protective effect of exclusive breastfeeding against viral infections, and the increased maternal contact of the breastfed infant compared with the infant receiving artificial or mixed feeding (41), and the high transmissibility of SARS-CoV, protective measures should be strictly observed for safeguarding the lactation process.

## Author Contributions

EV contributed to the conception, designed the review, and wrote the first draft. EV and GF collected the relevant literature and independently reviewed the studies, while GK further evaluated the studies where conflict was presented in the decision. EV, CM, LM, EB, and GK wrote sections of the manuscript. All authors contributed to the article and approved the submitted version.

## Conflict of Interest

The authors declare that the research was conducted in the absence of any commercial or financial relationships that could be construed as a potential conflict of interest.
